# Marked Increase in Diabetic Ketoacidosis Rates With Modest Increase in HbA1c Elevation at Diabetes Diagnosis in Swedish Children

**DOI:** 10.1111/apa.70726

**Published:** 2026-09-17

**Authors:** Frida Sundberg, Katarina Eeg‐Olofsson, Karin Åkesson

**Affiliations:** ^1^ Department of Paediatrics, Faculty of Medicine and Health Örebro University Örebro Sweden; ^2^ Swedish National Diabetes Register Centre of Registers, Region Västra Götaland Gothenburg Sweden; ^3^ Sahlgrenska University Hospital and Department of Molecular & Clinical Medicine University of Gothenburg Gothenburg Sweden; ^4^ Department of Paediatrics Ryhov County Hospital Jönköping Sweden; ^5^ Department of Biomedical and Clinical Sciences Linköping University Linköping Sweden

## Abstract

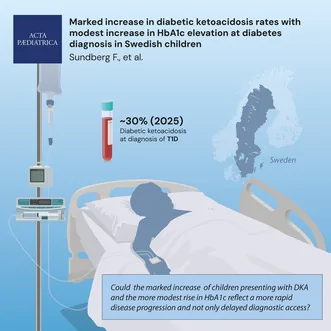

The proportion of children presenting with metabolic acidosis with pH < 7.3 at the diagnosis of type 1 diabetes (T1D) has increased in Sweden in recent years. Metabolic acidosis at diagnosis of T1D is almost exclusively diabetic ketoacidosis (DKA). In 2025, this proportion reached 30% for the first time since data collection for the quality register for diabetes care (Swediabkids/National Diabetes Register) began in the year 2000 (Table 1). In comparison, only 14%–17% of children with newly diagnosed T1D presented with DKA during the first 5 years of the register (2000–2004). Data from the first years of the registry need to be interpreted with caution as data collection was not complete at that time. Data from year 2007 and forward have almost complete national coverage. Data from 2007 to 2011 fit into the trend with any DKA in 17%–20% of children at diagnosis. When comparing the period 2007–2011 with the most recent 5‐year period (2021–2025), 4.3%–8.6% compared to 8.4%–10% of newly diagnosed children had severe DKA defined as pH < 7.1. Considering the COVID‐19 pandemic years (2020–2022), the proportion of children with DKA was higher in the post‐pandemic period than during and in the years preceding, the COVID‐19 pandemic [1].

**TABLE 1:** Number of children and proportion aged younger than 5 years, mean HbA1c (mmol/mol) and proportion of children with any DKA (pH < 7.3), severe DKA (pH < 7.1) and very severe DKA (pH < 7.0) at diagnosis per year.

Year	Reported children	Proportion aged < 5 years at diagnosis	Mean HbA1c (mmol/mol)	Proportion of children (%) with pH below		
	*N*	*%*		< 7.0	< 7.1	< 7.3
2000	723	18.0	90	2.0	4.3	17
2001	733	19.8	90	1.7	3.7	14
2002	777	17.5	Not reported	1.1	3.7	17
2003	805	17.9	Not reported	1.8	4.5	16
2004	826	18.4	93	1.9	4.7	17
2005	806	15.4	92	2.3	4.0	17
2006	790	18.0	94	1.6	4.4	22
2007	813	16.6	94	2.4	5.4	20
2008	767	17.3	92	2.2	4.3	17
2009	837	20.8	93	1.6	4.3	18
2010	825	19.9	93	2.6	5.2	19
2011	815	20.7	97	2.0	8.6	18
2012	863	20.0	92	1.1	3.9	19
2013	858	19.4	91	1.6	3.8	18
2014	888	19.4	93	3.2	5.7	21
2015	901	17.8	95	1.6	3.6	19
2016	945	17.8	94	3.4	5.4	25
2017	899	17.2	94	3.4	6.7	24
2018	780	17.2	95	3.3	5.7	24
2019	928	20.4	96	2.6	6.2	21
2020	701	16.3	98	3.4	7.0	26
2021	1098	20.9	97	4.6	8.7	28
2022	1044	22.3	98	4.1	8.4	28
2023	901	18.3	98	6.4	10	28
2024	1001	21.2	96	4.7	8.1	27
2025	933	18.7	98	4.2	9.0	30

In the public debate, this increase in DKA at diagnosis is often attributed to delayed access to diagnostic evaluation by health‐care personnel (HCP), a phenomenon studied by Wersäll in a cross‐sectional analysis. Wersäll reported that HCP‐delayed diagnosis was associated with a higher risk of DKA in children with newly diagnosed T1D [2]. However, as this was a cross‐sectional study, no conclusions can be drawn regarding whether this phenomenon has increased over time.

If delayed diagnosis were the primary cause of the rising proportion of children presenting with DKA, a corresponding increase in HbA1c at diagnosis would be expected. Mean HbA1c values during the period 2007–2011 were 92–97 mmol/mol, compared with 96–98 mmol/mol in the most recent five reported years. Although this represents an increase, it remains uncertain whether it is proportional to the marked rise in the DKA frequency.

One possible explanation for the discrepancy between the marked increase in the proportion of children presenting with any DKA or severe DKA and the more modest rise in HbA1c could be a more rapid progression of the disease itself, rather than delayed diagnostic access.

The epidemiology of type 1 diabetes in children has changed substantially over the past 40 years. The incidence in children younger than 15 years doubled between 1978 and 2019 with the most prominent increase in year 1978–2000. The incidence in children aged 0–4 years in 2019 was similar to the total paediatric incidence in 1978 [3]. However, the proportion of children being younger than 5 years at diagnosis has varied between 17% and 21%, with few exceptions, in year 2000–2025 (Table 1). The increased proportion of children presenting with DKA at diagnosis may fit within a broader pattern of a more aggressive disease course in T1D.

These observations, based on the annual Swediabkids/National Diabetes Register reports as they are published, require thorough analysis of the data, including age distribution and associations of HbA1c with age before firm conclusions can be drawn. Regardless of the underlying cause of the increased proportion of DKA at diagnosis, it remains essential clinical advice to measure plasma glucose immediately in any child with symptoms suggestive of diabetes. However, if data analyses support the presence of a more aggressive disease process, further research into modifiable risk factors for disease progression will be warranted.

## Author Contributions

**Katarina Eeg‐Olofsson:** writing – review and editing. **Karin Åkesson:** writing – review and editing. **Frida Sundberg:** conceptualization, writing – original draft, writing – review and editing.

## Funding

The authors have nothing to report.

## Conflicts of Interest

F.S., K.Å. reports personal lecture fees from Sanofi, no conflicts of interest relevant for this manuscript. K.E.‐O.: reports personal lecture fees and/or honoraria for consulting from Sanofi, Eli Lilly, Novo Nordisk, Astra Zeneca and Abbott diabetes care, all outside the submitted work.

## Data Availability

The data that support the findings of this study are available in ndr.registercentrum.se. These data were derived from the following resources available in the public domain Annual reports: ndr.registercentrum.se.
